# Perinatal brain damage: The term infant

**DOI:** 10.1016/j.nbd.2015.09.011

**Published:** 2016-08

**Authors:** Henrik Hagberg, A. David Edwards, Floris Groenendaal

**Affiliations:** a Centre for the Developing Brain, Department of Perinatal Imaging and Health, Division of Imaging and Biomedical Engineering, King's College London, King's Health Partners, St. Thomas' Hospital, London, SE1 7EH, United Kingdom; b Perinatal Center, Dept of Clinical Sciences, Sahlgrenska Academy, University of Gothenburg, Sweden; c Department of Neonatology, Wilhelmina Children's Hospital, University Medical Center Utrecht, Utrecht, The Netherlands; d Brain Center Rudolf Magnus, University Medical Center Utrecht, Utrecht, The Netherlands

**Keywords:** Perinatal, Brain injury, Neonatal encephalopathy, Hypoxia–ischaemia, Hypothermia, Neuroprotection, Diagnosis

## Abstract

Perinatal brain injury at term is common and often manifests with neonatal encephalopathy including seizures. The most common aetiologies are hypoxic–ischaemic encephalopathy, intracranial haemorrhage and neonatal stroke. Besides clinical and biochemical assessment the diagnostic evaluation rely mostly on EEG and neuroimaging including cranial ultrasound and magnetic resonance imaging. The mechanisms underlying hypoxic–ischaemic brain injury are only partly understood but include excitotoxicity, mitochondrial perturbation, necrosis/apoptosis and inflammation. Neuroprotective treatment of newborns suffering from hypoxic–ischaemic encephalopathy with hypothermia has proven effective and has been introduced as a clinical routine. Ongoing studies are exploring various add-on therapies including erythropoietin, xenon, topiramate, melatonin and stem cells.

## 1 Introduction

Perinatal brain injury in the term infant is common in both developed and underprivileged countries. Almost all forms of perinatal brain injury will result into neonatal encephalopathy of which seizures and reduced reactivity are the most frequent clinical findings.

Neonatal encephalopathy occurs in 1–3 per 1000 infants born at term and is most commonly due to hypoxia–ischaemia (Glass et al., 2009, Ronen et al., 1999, Vasudevan and Levene, 2013). Risk factors for hypoxic–ischaemic encephalopathy include a gestational age above 41 weeks, prolonged membrane rupture, abnormal cardiotocography, thick meconium, sentinel event, shoulder dystocia, tight nuchal cord, failed vacuum (Martinez-Biarge et al., 2013), and white matter injury, which was associated with hypoglycaemia and mutations in the MTHFR gene resulting in elevated levels of plasma homocysteine (Harteman et al., 2013a).

Encephalopathy can also result from perinatal/neonatal stroke. For cerebral venous sinus (sinovenous) thrombosis dehydration is added to the risk factors for neonatal stroke (Nwosu et al., 2008), which include maternal risk factors such as infertility, primiparity, maternal fever, meconium-stained amniotic fluid, chorioamnionitis, pre-eclampsia and intrauterine growth retardation. Complicated deliveries, both instrumental and emergency caesarean section, low Apgar scores and hypoglycaemia are more frequently observed in infants with neonatal stroke. Prothrombotic factors are more often observed. Congenital heart disease carries also a higher risk of neonatal stroke (van der Aa et al., 2014).

Seizures are common during neonatal encephalopathy, but may also be due to other causes. They are commonly seen after neonatal stroke, and may be the only clinical manifestation of this condition. The incidence of seizures in the newborn period can only be properly estimated with (continuous) EEG monitoring (Murray et al., 2008), but the aetiology of seizures in (near) term infants using extensive imaging, metabolic and genetic examinations has been studied recently (Weeke et al., 2015). The most common aetiologies identified were hypoxic–ischaemic encephalopathy (46%), intracranial haemorrhage (12.2%), perinatal arterial ischaemic stroke (10.6%), cerebral sinovenous thrombosis (2.9%), metabolic disorders including hypoglycaemia (9%), central nervous system infection (7.1%), cerebral dysgenesis (2.9%), and genetic disorders, predominantly benign familial neonatal seizures (2.1%).

### 1.1 Pathophysiology of hypoxic–ischaemic (HI) brain injury

#### 1.1.1 Phases of brain injury

The development of brain injury can be divided into different stages (Fleiss and Gressens, 2012). Cerebral HI, of sufficient severity to deplete tissue energy reserves (primary phase), is often followed by near-complete restoration of glucose use (Gilland and Hagberg, 1996), mitochondrial respiration (Gilland et al., 1998) and high-energy phosphates (Penrice et al., 1997) after reperfusion and reoxygenation (latent phase). Thereafter, a secondary phase occurs encompassing a decrease in high-energy phosphates accompanied by cell demise often referred to as secondary brain injury or secondary energy failure (Azzopardi et al., 1989, Blumberg et al., 1997, Lorek et al., 1994).Several interventions have been shown to attenuate secondary brain injury in experimental models (Hagberg et al., 2014, Hagberg et al., 2015) and hypothermia has proven effective also in the clinical setting (Azzopardi et al., 2014). Less is known about mechanisms of long-term cell injury and repair (tertiary phase) (Fleiss and Gressens, 2012), and so far treatment strategies have focused on the latent phase. However, recent findings from experimental studies indicate that the therapeutic window could be extended beyond the secondary phase, and interventions targeting late stages of inflammation or post-lesion repair may be a possibility (see below).

#### 1.1.2 Mechanisms of secondary brain injury

The specific mechanisms of secondary damage are only partly understood, but excitotoxicity, mitochondrial impairment, intracellular regulation of Ca^2+^, oxygen and nitrosative stress, deficiency of trophic factors and inflammation are all implicated in the process (Hagberg et al., 2014, Hagberg et al., 2015, Thornton et al., 2012). Furthermore, both accidental and regulated (e.g. apoptosis, necroptosis) cell death appears critical in the execution phase of cellular demise (Thornton and Hagberg, 2015, Johnston, 2005).

##### 1.1.2.1 Excitotoxicity

Glutamate and aspartate are believed to be the main excitatory amino acids (EAAs) in the brain (Johnston, 2005). Besides their excitatory actions they are also known to exert toxic effects (excitotoxicity) on neurons (Johnston, 2005) and oligodendroglial precursors (OPCs) (Follett et al., 2004, Salter and Fern, 2005) in the CNS. Both N-methyl-d-aspartate (NMDA) and α-amino-3-hydroxy-5-methyl-4-isoxazolepropionic acid (AMPA)/kainate receptors are expressed on neurons and oligodendroglial precursors (preferentially on somata) in vulnerable areas of grey and white matter in the immature brain and NMDA receptors are expressed on microglia (Kaindl et al., 2012, Tahraoui et al., 2001). There is evidence for a role of EAAs in HI brain injury. Extracellular concentrations of EAAs, and to some extent glycine, increase extracellularly during neonatal HI in mixed grey and white matter of foetal sheep (Hagberg et al., 1987) and is followed by a secondary increase during reflow (Thoresen et al., 1997). EAAs also increase markedly in the CSF of newborns with neonatal encephalopathy and the levels are associated with the degree of encephalopathy and short-term outcome (Hagberg et al., 1993). Blocking NMDA receptors before or after HI in experimental models reduces subsequent neuronal damage (Hagberg et al., 1994)(Table 1) and during in vitro ischaemia, NMDA receptor activation results in Ca^2**+**^-dependent injury of oligodendroglial processes (Salter and Fern, 2005). AMPA blockade reduces grey and white matter damage when given after HI or an excitotoxic insult (Follett et al., 2004, Hagberg et al., 1994, Sfaello et al., 2005) (Table 1).

**Fig. 1:**
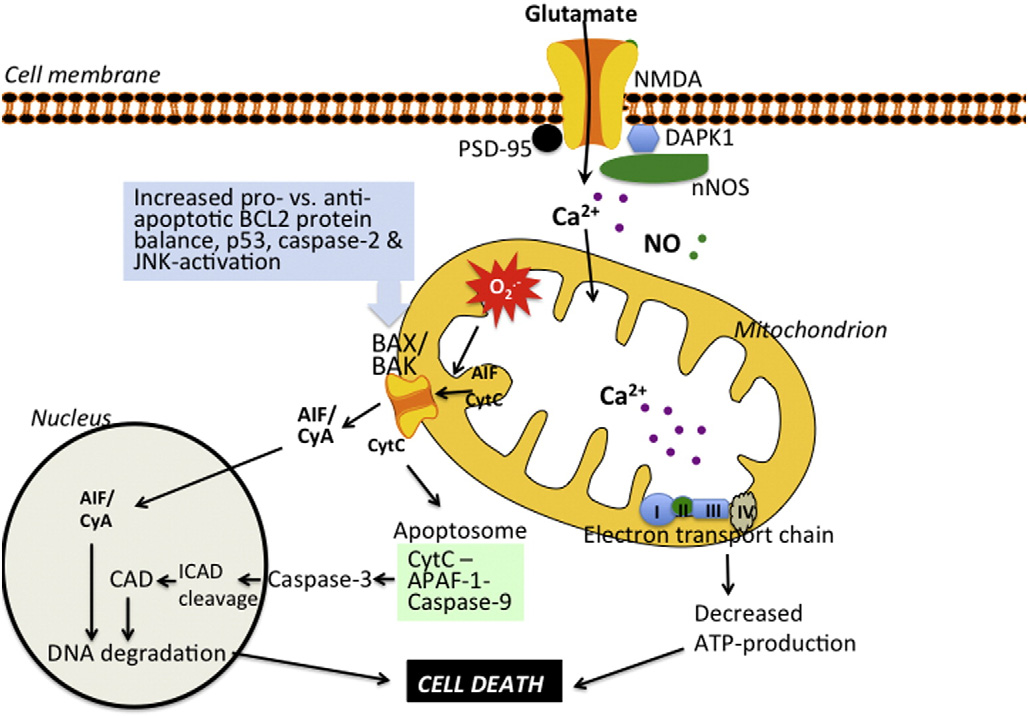
Role of mitochondria in hypoxic–ischaemic brain injury**.** During early reperfusion, extracellular glutamate activates NMDA receptors leading to intracellular accumulation of Ca^2+^ and nitric oxide (NO). NO and mitochondrial Ca^2+^ accumulation (Ca^2 +^ dysregulation) elicit production of reactive oxygen species (superoxide, O2^.-^) which induces respiratory suppression, and contributes to translocation of cytochrome c (CytC) and apoptosis-inducing factor (AIF) from the inner mitochondrial membrane/cristae to the intermembrane space. An increase of pro-apoptotic compared with anti-apoptotic BCL2 family proteins, activation of caspase-2, and interaction of p53 with the outer mitochondrial membrane leads to mitochondrial permeabilisation. The subsequent release of CytC triggers a cascade including assembly of the apoptosome and activation of executional caspases and, leading to degradation of DNA and essential proteins, and resulting in cell death. AIF binds to cyclophilin A (CyA) and the complex translocates to the nucleus and induces chromatinolysis. PSD-95 = postsynaptic density protein 95. nNOS = neuronal NO synthase. CAD = caspase-activated DNase. ICAD = inhibitor of CAD. APAF1 = apoptic peptidase activating factor 1.

Overflow of extracellular glutamate activates both extrasynaptic and intrasynaptic NMDA receptors (Fig. 1). The extrasynaptic component probably predominates under HI conditions, leading to intracellular accumulation of Ca^2+^ and substantial production of nitric oxide (NO), both of which exert stress on mitochondria (Hagberg et al., 2014, Hardingham and Bading, 2010). The mechanism underlying toxic effects of extrasynaptic NMDA receptors on mitochondria is unknown, but NO synthase, mitogen-activated protein kinases (particularly c-jun N-terminal kinases), and calpains probably play a part (Blomgren et al., 2001, Ferriero et al., 1996, Hardingham and Bading, 2010, Nijboer et al., 2013, Pirianov et al., 2007). Intrasynaptic NMDA receptors can also mediate toxic effects (Wroge et al., 2012) but paradoxically inhibition of synaptic NMDA receptors can also deprive the cell of an endogenous salvage pathway and trigger widespread apoptotic cell death in the immature brain (Ikonomidou et al., 1999). Possibly this adverse effect can be circumvented through selective blocking of extrasynaptic NMDA receptors. For example, inhibitors of extrasynaptic NMDA receptors that act on postsynaptic density protein 95, DAPK1, or NMDA receptor 2B have been suggested as promising candidates (Tu et al., 2010, Cook et al., 2012a, Cook et al., 2012b, Nair et al., 2013).

##### 1.1.2.2 Mitochondrial impairment and Ca^2 +^ regulation

During HI, electron flow through the electron transport chain slows down due to lack of oxygen, the H^+^ gradient across the inner membrane decreases and ATP production drops (Fig. 1). Under such conditions combined with high cytosolic concentrations of Ca^2+^ and NO (due to activation of NMDA receptors and opening of voltage-dependent Ca^2 +^ channels) the intramitochondrial Ca^2+^ increases (Puka-Sundvall et al., 2000). The accumulation of Ca^2+^ within the mitochondrial matrix activates mitochondrial phosphatases leading to hyperactivation of cytochrome c oxidase and cytochrome c and pathological hyperpolarisation of the mitochondrial membrane potential during the early recovery phase after the insult (Sanderson et al., 2013, Starkov and Fiskum, 2003). Mitochondrial inner membrane hyperpolarisation, in turn, triggers excessive production of reactive oxygen species (ROS) by complexes 1 and 3 (Piantadosi and Zhang, 1996, Sanderson et al., 2013, Starkov and Fiskum, 2003) which in combination with an increase of NO (Ferriero et al., 1996) result in suppression of mitochondrial respiration during the early secondary phase of brain injury after HI (Gilland et al., 1998, Starkov and Fiskum, 2003). Furthermore, ROS oxidise cardiolipin, which in turn releases cytochrome c from the inner mitochondrial membrane. ROS have also been proposed to oxidise Optic Atrophy 1, leading to opening of the proximal part of the cristae thereby allowing diffusion of cytochrome c into the intermembrane space (Ramonet et al., 2013). Apoptosis-inducing factor (AIF) is also believed to be attached to the inner mitochondrial membrane under physiological conditions. Apoptosis-inducing factor can also translocate to the intermembrane space in response to oxidative stress, activation of poly(ADP-ribose) polymerase (PARP)1, and activation of proteases (Modjtahedi et al., 2006). This translocation is a prerequisite for the subsequent transfer of AIF to the cytosol and nucleus after mitochondrial permeabilisation (Modjtahedi et al., 2006).

##### 1.1.2.3 Mitochondrial permeabilisation and apoptotic cell death

At a certain threshold of mitochondrial perturbation the organelle undergoes outer membrane permeabilisation and apoptotic cell death is executed. Proapoptotic proteins (cytochrome c and AIF) are released from mitochondria, the apoptosome forms, and downstream executioner caspases (particularly caspase-3) are activated after HI (Cheng et al., 1998, Galluzzi et al., 2009, Northington et al., 2011a) resulting in DNA fragmentation and cell death. Pathways dependent on AIF (Zhu et al., 2007) and caspases (Hu et al., 2000) seem to be more strongly activated in the immature brain than in the adult brain (Hu et al., 2000, Zhu et al., 2005), and mitochondrial permeabilisation seems to mark the point of no return in HI injury of the immature brain. Such a concept is also supported by studies showing that apoptotic morphologies are predominant phenotypes after brain injury in the immature brain (Edwards and Mehmet, 1996) and that treatment with hypothermia reduces apoptotic (rather than necrotic) cell death (Edwards et al., 1995).

The molecular mechanism of mitochondrial permeabilisation after HI is still incompletely understood. In the adult brain, opening of a cyclophilin D (*Ppid*) dependent permeability transition pore is critical for development of ischaemic injury (Schinzel et al., 2005), whereas *Ppid* gene deficiency has the opposite effect in immature models of HI (Wang et al., 2009). Instead mitochondrial permeabilisation in the immature CNS after HI depends on BAX–BAK-dependent pore formation. Hence, BAX-inhibitory peptides (Wang et al., 2009, Wang et al., 2010) and BAX gene deficiency (Gibson et al., 2001) substantially protect the immature brain (Table 1). Several proapoptotic and antiapoptotic members of the BCL2 protein family seems to be crucial in triggering BAX–BAK dependent mitochondrial permeabilisation (Ness et al., 2006). Additionally, interaction of the tumour suppressor p53 and xcaspase-2 with BCL2 members at the outer mitochondrial membrane contributes to mitochondrial permeabilisation (Carlsson et al., 2011, Nijboer et al., 2011). The above mentioned interventions targeting BAX–BAK-dependent mitochondrial permeabilisation directly or indirectly, are neuroprotective (Table 1), suggesting a central role for mitochondria in directing apoptotic cell death in the developing brain.

##### 1.1.2.4 Necrotic/necroptotic cell death

Another major form of cell death is necrosis, which initially was considered to be accidental or uncontrolled, characterised by cellular swelling and membrane rupture. However, it is now clear that at least some forms of necrosis result from a regulated series of events referred to as regulated or programmed necrosis which occurs in an environment that is either dramatically depleted of ATP or in which caspases are inhibited (Galluzzi et al., 2011, Vanden Berghe et al., 2014). One form of regulated necrosis, “necroptosis”, depends on activation of receptor-interacting kinase (RIP)3, mixed lineage kinase domain-like (MLKL) and sometimes RIP1. In a neonatal mouse model of HI, administration of necrostatin-1 (RIP1 inhibitor) attenuated progression of injury and appeared to shift cell death towards apoptosis (Northington et al., 2011b). Necrostatin-1 also decreased the accumulation of oxidants, prevented the decline in mitochondrial complex I activity and improved ATP levels after HI (Chavez-Valdez et al., 2012), supporting the concept that execution of necroptosis in the immature brain depends on mitochondria at least partly (Thornton and Hagberg, 2015).

##### 1.1.2.5 Inflammation

HI or stroke induces an inflammatory reaction in the immature brain (Hagberg et al., 2015, Derugin et al., 2000). Cells belonging to the innate or adaptive immune system are activated including microglia/macrophages (McRae et al., 1995, Tahraoui et al., 2001), polymorphonuclear cells (Bona et al., 1999, Palmer et al., 2004), lymphocytes (Bona et al., 1999, Winerdal et al., 2012), NK-cells (Bona et al., 1999, Winerdal et al., 2012) and mast cells (Patkai et al., 2001, Jin et al., 2009). The accumulation of cells is accompanied by altered expression of innate immune receptors (including Toll-like receptors (TLRs) and Nodd-like receptors (NLRs) cytokines, chemokines, ROS, excitatory amino acid agonists, and death receptor agonists including TNF, FasL, RANKL, TRAIL and TWEAK which could contribute to cell death (Hagberg et al., 1996, Hedtjarn et al., 2002, Hedtjarn et al., 2004, Ivacko et al., 1997, Kichev et al., 2014, Mallard et al., 2009, Silverstein et al., 1997). In parallel to these alterations, the blood–brain-barrier undergoes transient opening at 6–24 h after HI (Ek et al., 2015). The initial inflammatory response is thought to depend on activation of innate immune receptors. TLRs are induced during recovery from neonatal HI, and knocking out *Tlr2* in mice provides neuroprotection (Stridh et al., 2011). Indirect evidence suggests that the inflammasome and NOD-like receptors also are involved as IL-1 production is increased after HI (Hagberg et al., 1996) and injury is attenuated by the IL-1 receptor antagonist IL-1RA (Hagberg et al., 1996) or by caspase-1 or IL-18 gene deficiency (Hedtjarn et al., 2002, Liu et al., 1999). There is further support that the early proinflammatory phase aggravates injury after HI, as inhibition of platelet-activating factor (Liu et al., 1996) and the complement C1q reduce HI injury (Ten et al., 2005). The inflammatory reaction is highly dependent on context and time. For example, the microglial/macrophage responses exert beneficial or detrimental effects depending on the phenotype. Microglia are likely to assume distinct functional phenotypes after HI in analogy with the M1, M2a and M2b phenotypes suggested in other models (Chhor et al., 2013, Colton and Wilcock, 2010). There are multiple neuroprotective interventions that have been proven effective in experimental and clinical studies that mainly or partly modify the immunoinflammatory response (Table 1).

#### 1.1.3 Tertiary phase of brain injury

As mentioned previously, experimental and human data strongly support the fact that hypothermia is neuroprotective in term infants if introduced within the first 6 h (see below) after birth. However, the therapeutic window may be much longer than that. Some studies suggest that it may be possible to intervene during the delayed inflammatory phase and improve post-lesional plasticity (Fleiss and Gressens, 2012). An intriguing clinical study has recently shown that EPO, when given on an average of 24 h after birth, had very significant neuroprotective effects on human term infants with neonatal encephalopathy (Zhu et al., 2009). Genetic upregulation of the complement factor C3a 6 h–7 days after HI significantly improved neurogenesis, memory functions and reduced brain injury in 9-day-old mice (Jarlestedt et al., 2013). Most impressively, intracerebral or intranasal administration of mesenchymal stem cells as late as 3–10 days following HI reduces lesion size and substantially improves memory and sensory-motor functions after HI in newborn rodents (Donega et al., 2013, van Velthoven et al., 2010).

#### 1.1.4 Diagnosis of brain injury

Many tests are performed to diagnose the extent and severity of perinatal brain damage (Table 2).

#### 1.1.5 Clinical assessment

##### 1.1.5.1 Apgar score

The Apgar score has been used as one of the first tests used to assess the severity of perinatal asphyxia. At present the Apgar score is no longer considered to be a marker of perinatal asphyxia and encephalopathy, although the score still predicts outcomes at a population level and the prognosis of term neonates with a very low Apgar score at 5 or 10 min after birth remains poor (Bonifacio et al., 2015, Casey et al., 2001, Laptook et al., 2009, Sarkar et al., 2010).

##### 1.1.5.2 Clinical examination

Clinical scoring systems have been used widely to assess the severity of neonatal encephalopathy. The most commonly used is the Sarnat score (Sarnat and Sarnat, 1976).This score combines information of clinical examinations and EEG and grades encephalopathy as mild (grade I), moderate (grade II) or severe (grade III). Outcome of infants with mild encephalopathy is uniformly good, whereas 25% of infants with moderate and almost 100% of infants with severe encephalopathy die or show severe impairments. Alternatives which are used nowadays are the Thompson score system (Thompson et al., 1997), which uses more clinical items than the Sarnat score, and does not need electrophysiological assessment. These scores have little value in diagnosing the cause of the encephalopathy.

#### 1.1.6 Biochemical assessment

##### 1.1.6.1 Blood gas values

Blood gas values have been reported as better indices of perinatal asphyxia than the Apgar score. Indeed, a relation between pH values and the occurrence of severe encephalopathy has been demonstrated (Goodwin et al., 1992, Lavrijsen et al., 2005). Nevertheless, many patients with a pH value of the umbilical artery below 7.0 do not show any signs of encephalopathy. Blood gas values remain associated with outcome in the era of therapeutic hypothermia (Bonifacio et al., 2015, Wayock et al., 2014).

##### 1.1.6.2 Biomarkers

Biochemical biomarkers have been examined during recent decades, but none of the biomarkers tested were able to predict the presence of brain injury (Serpero et al., 2013), although they might be useful in specific diseases (Tonni et al., 2014).

#### 1.1.7 Electrophysiology

##### 1.1.7.1 Evoked potentials and amplitude-integrated EEG

Evoked potentials have some value in determining the severity of a hypoxic–ischaemic insult in an encephalopathic term neonate (Eken et al., 1995, van Laerhoven et al., 2013). They are indicative of the severity of brain injury, and are predictive of outcome, but are less commonly used since the introduction of aEEG.

EEG, in particular amplitude-integrated continuous EEG (aEEG) is commonly used in encephalopathic infants, in particular in Europe (Boylan et al., 2013), and can help detect seizures. aEEG abnormalities (seizures and a suppressed background pattern) have high predictive values towards an adverse outcome after perinatal asphyxia (Toet et al., 1999, van Laerhoven et al., 2013), both in normothermic infants as well as during therapeutic hypothermia (Thoresen et al., 2010).

#### 1.1.8 Near-infrared spectroscopy

Near-infrared spectroscopy (NIRS) is applied in term neonates with perinatal asphyxia to measure brain oxygen saturation. In severe encephalopathy following perinatal asphyxia very high oxygen saturation levels have been encountered, coinciding with low aEEG background patterns. This suggests lack of oxygen consumption in these affected infants, and helps predict a poor outcome (Lemmers et al., 2013).

#### 1.1.9 Neuroimaging

##### 1.1.9.1 Cranial ultrasound

Cranial ultrasound is a useful technique to detect major abnormalities including large intraventricular, subdural, or cerebellar haemorrhage, ischaemic infarctions, and calcifications (Steggerda et al., 2009). In term neonates major changes to the basal ganglia and thalamus can sometimes be identified during the first days after an acute hypoxic–ischaemic insult, although ultrasound lacks sensitivity and specificity in predicting outcomes in neonatal encephalopathy (Eken et al., 1994).

##### 1.1.9.2 Magnetic resonance imaging and spectroscopy

MRI is the most sensitive technique to detect abnormalities in the brain, but specific and neonatally-adapted sequences are required to obtain maximal information. In addition to standard sequences diffusion weighted imaging is needed to detect cytotoxic oedema within the first week after the hypoxic–ischaemic insult, susceptibility weighted sequences may be needed to detect small haemorrhages, and MR angiography and venography are required to detect abnormalities in blood vessels.

MRI abnormalities have a high predictive value for an abnormal outcome, also in the era of therapeutic hypothermia following perinatal asphyxia (Alderliesten et al., 2011, Rutherford et al., 2010). MRI is also useful to detect other acquired brain injuries such as lesions due to trauma, sinovenous thrombosis and arterial infarctions.

Proton MR spectroscopy of the basal ganglia and thalamus has a reasonably high predictive value for an abnormal neurodevelopmental outcome (Alderliesten et al., 2011).Even with dedicated MRI systems brain injury may be less well detected than through histology (Alderliesten et al., 2013).

### 1.2 Outcome of perinatal brain damage

Outcome of moderate-to-severe encephalopathy following perinatal asphyxia is still a matter of concern (Jacobs et al., 2013). Although the introduction of therapeutic hypothermia has reduced the incidence of poor outcome from approximately 60% to 45%, death or neuromotor impairment is still common.

The most severe forms of perinatal brain damage will lead to cerebral palsy. When the basal ganglia and thalamus are involved as in kernicterus or severe, acute perinatal asphyxia the cerebral palsy will be of the dyskinetic quadriplegia type, whereas unilateral spastic cerebral palsy will be seen after unilateral infarcts such as middle cerebral artery infarct.

After white matter injury long-term cognitive and behavioural consequences are more commonly seen (Van Kooij et al., 2010), although the outcome is not invariably poor (Harteman et al., 2013b). The specific pattern of MRI changes after perinatal asphyxia and the outcome may be dependent on several associated factors, including hypoglycaemia, genetic factors, and placental pathology (Harteman et al., 2013c, Martinez-Biarge et al., 2013).

Recent papers suggest that milder encephalopathy may result in subtle changes in neurodevelopment later in life (Van Kooij et al., 2010).

### 1.3 Treatment of perinatal brain damage at term

The goal of neuroprotective therapy is to prevent injury progression, salvage and protect the cells that would otherwise be injured or die, repair the injured cells, and enhance neurogenesis with the long-term goal of improving neurodevelopmental outcomes. Much has been learnt from animal and adult studies on brain injury looking at potential treatment strategies (Table 1), and allowing for potential translation into clinical research and trials. The success of moderate hypothermia in term hypoxic–ischaemic encephalopathy has provided proof of concept that neural rescue by intervention *after* the hypoxic–ischaemic insult is possible.

#### 1.3.1 Hypothermia

Therapeutic hypothermia using a targeted temperature reduction regimen in which there are sequential phases of moderate cooling, maintenance and slow rewarming is the standard of care for neonatal hypoxic–ischaemic injury in term infants (Azzopardi et al., 2009, Gluckman et al., 2005, Shankaran et al., 2005, Zhou et al., 2010). Six major clinical trials in neonates – The Cool Cap, NICHD, TOBY, neo.nEURO.network Trial, the China Study Group, and ICE trials – all showed either overall benefit of cooling for HIE or benefit within subgroups (Azzopardi et al., 2009, Gluckman et al., 2005, Jacobs et al., 2011, Shankaran et al., 2005, Simbruner et al., 2010, Zhou et al., 2010). Meta-analysis showed that therapeutic hypothermia reduced death or disability in moderate encephalopathy at 18 months with a risk ratio of 0.62 [95% confidence interval (CI) 0.50–0.77] and in severe encephalopathy a risk ratio of 0.88 (95% CI 0.78–0.99) (Edwards et al., 2010). With cooling, 6 out of 10 infants with moderate to severe insult have a normal outcome at 2 years of age. The NICHD Whole Body Cooling trial and TOBY trials show that the reduction of death and disability noted at 18 months persist to childhood (Azzopardi et al., 2014, Shankaran et al., 2012), although there were some differences: in the NICHD trial the effect was through reduced death, while in TOBY there was no difference in the death rate, rather a reduction in impairment in survivors. Adverse events (AEs) such as arrhythmias, bleeding, skin effects due to cooling, hypotension, persistent pulmonary hypertension and infection were minimal in the clinical trials (Shankaran et al., 2008), but in post-introduction surveillance subcutaneous fat necrosis was noted in 1% of the cases registered with the UK cooling register (Strohm et al., 2011).

Hypothermic neural rescue is now widely practised as standard of care in developed countries. The American Heart Association and the National Institute for Health and Clinical Excellence (UK) recommend that therapeutic hypothermia be commenced as post resuscitation care for term infants meeting the criteria used in published clinical trials and the British Association of Perinatal Medicine published guidelines for neonatal units and networks to standardise hypothermia therapy (Adams et al., 2010, Kattwinkel et al., 2010, NICE interventional procedure guidance IPG347, 2010). The economic benefit of cooling calculated from increase in economically active survivors and reduction in costs of care of disabilities is already in excess of £100 million (Edwards et al., 2013).

Further research into hypothermia is ongoing. Although cooling has been shown to be beneficial in high resource settings, this has not been shown in low resource settings and a clinical trial looking at cooling in a low resource setting is currently underway (ISRCTN89547571). Although in the cooling trials, hypothermia was initiated at a median age of 4 h and continued for 72 h, it is not known whether delay in cooling or continuing hypothermia may also be beneficial. A clinical trial: Late Hypothermia for HIE (NCT00614744) is currently ongoing where term infants between 6 and 24 h of age will be recruited and hypothermia will be continued for 96 h.

#### 1.3.2 Novel and experimental treatments

Although hypothermia has been successful, the rate of death or moderate to severe disability in infants with moderate to severe HIE after cooling is 46% (95% CI 40–53%) (Edwards et al., 2013). There is still an urgent need for additional therapies to improve outcomes and achieve maximal neuroprotective effect. A number of therapies are in the preclinical phase or currently undergoing phase 2 and phase 3 clinical trials.

##### 1.3.2.1 Erythropoeitin (EPO)

EPO is a growth factor with immunomodulatory, vasogenic and proangiogenic effects, best known as a regulator of red cell production (Chong et al., 2002, Wang et al., 2004, Villa et al., 2003). There is increasing evidence that EPO has a neuroprotective effect through both direct neuronal receptor mediated interaction and indirect effects (Edwards et al., 2013). It has been shown in many animal models that EPO decreases apoptosis (Digicaylioglu and Lipton, 2001), excitotoxicity (Kellert et al., 2007), glutamate toxicity (Kawakami et al., 2000) and inflammation (Sun et al., 2005). EPO has been shown to improve cognitive outcome (Kumral et al., 2004, Sola et al., 2005a, Sola et al., 2005b) with evidence of neurogenesis (Plane et al., 2004, Wang et al., 2004) (Table 1). Delayed treatment 24 h after the insult has also shown to be neuroprotective (Sun et al., 2005). Five clinical trials looking at safety and efficacy of EPO as a potential neuroprotective therapy in neonates have been published: two in preterm infants and three in term infants (Elmahdy et al., 2010, Fauchere et al., 2008, Juul et al., 2008, Zhu et al., 2009, Wu et al., 2012), however only 2 of these studies looked at outcome measures. In both the clinical trials, the patient numbers were small, decreased length of follow up and a lack of intention to treat analysis.

##### 1.3.2.2 Xenon

Xenon is an anaesthetic noble gas which is a non-competitive antagonist of the NMDA subtype of the glutamate receptor (Franks et al., 1998) and which also inhibits the apoptotic pathway through Bcl-XL and Bcl-2 (Ma et al., 2007) (Table 1), inducing the expression of HIF 1α and its downstream effectors erythropoietin and vascular endothelial growth factor (Kilic et al., 2005). It is highly neuroprotective in a variety of models of acute neuronal injury (Dingley et al., 2006, Ma et al., 2003, Ma et al., 2006, Wilhelm et al., 2002). There are a number of clinical trials ongoing looking at the neuroprotective effects of xenon. There is a study in adults looking at the synergistic neuroprotection of xenon and hypothermia following cardiopulmonary arrest (NCT00879892). The COOLXENON-2 trial is studying the effect in term HIE infants of inhaled xenon 50% for 18 h and 72 h of cooling as standard with normalisation of aEEG and MRI at term weeks of age as primary outcome (NCT01545271). The TOBYXe Study is recruiting term moderate to severe HIE neonates to 30% inhaled xenon for 24 h starting within 12 h of birth along with therapeutic hypothermia for 72 h as standard treatment (NCT00934700); this trial has already reported an anticonvulsant effect of xenon in encephalopathic infants (Azzopardi et al., 2013). Results of these studies are awaited.

##### 1.3.2.3 Topiramate (TPM)

TPM is an anticonvulsant with neuroprotective properties in animal models (Costa et al., 2006, Guerrini and Parmeggiani, 2006, Shank et al., 2000, Yang et al., 1998) (Table 1), acting by inhibiting the glutamate receptors AMPA and kainate receptors along with blockade of the sodium channels, high voltage calcium channels and mitochondrial permeability transition pore (MPTP) (Costa et al., 2006, Kudin et al., 2004, Zona et al., 1997). So far, no clinical trials have been published proving the neuroprotective action of TPM with or without hypothermia in newborns with HIE. There is a clinical trial currently running on safety and efficacy of topiramate in neonates with hypoxic ischaemic encephalopathy treated with hypothermia (NeoNATI) (NCT01241019) where 10 mg/kg of topiramate is being administered orally in the first 3 days of life with neurologic and neuroradiologic outcome at 24 months.

##### 1.3.2.4 Melatonin

Melatonin (N-acetyl-5-methoxytriptamine) is a naturally occurring hormone secreted by the pineal gland which influences the sleep–wake cycle (Reiter, 1991) and has neuroprotective properties of melatonin probably both through the MT1 and MT2 receptors (Dubocovich et al., 2010) and through direct receptor-independent free radical scavenging actions (Tan et al., 2002).

There is strong experimental evidence of a neuroprotective effect of melatonin (Table 1). Husson et al. showed that melatonin at lower doses decreases white matter lesions but not cortical lesions while antioxidants like N-acetylcysteine appears to protect both lesions (Husson et al., 2002). In a recent study in the perinatal asphyxia piglet model, Robertson et al. showed that the Lac/Cr and Lactate/N acetyl aspartate were significantly lower in the piglets who received melatonin along with moderate hypothermia (Robertson et al., 2013). Clinical trials are awaited.

##### 1.3.2.5 Stem cell therapy

Stem cells are multipotent cells, capable of long-term self-renewal and possess extensive proliferative capacity. A variety of stem cell approaches are proposed for treatment of HIE and include neural stem cells, mesenchymal stem cells, cord blood cells and foetal grafts.

In adult animals neural stem cell (NSC) transplantation after brain injury results in integration of the cells into the injured tissue (Hicks et al., 2007), decreased volume loss (Hoehn et al., 2002) and improved functional recovery (Hicks et al., 2007). In the neonatal models, implantation of NSCs also resulted in migration to injured areas with differentiation into neuronal cell types (Park et al., 2006a, Park et al., 2006b, Sato et al., 2008). In hypoxic ischaemic rats cells transplanted 3 days after the insult initially proliferated with some cells surviving for a prolonged period (Obenaus et al., 2011). There are currently no clinical trials in neonates using NSCs.

Mesenchymal stem cells (MSCs) have the ability to differentiate not only into cells of mesodermal lineage but also neurons as well. In neonatal HI, transplantation of MSCs from umbilical cord blood resulted in functional improvement with decrease in activated microglia (Pimentel-Coelho and Mendez-Otero, 2010), and Kaneko et al. have also shown in rat neurons subjected to HI injury, combination of MSCs with hypothermia significantly improved the survival of the neurons (Kaneko et al., 2012). As these cells are available in abundance and ethically acceptable, there has been an interest in umbilical cord blood cell transplantation, and there is currently a safety and feasibility study using autologous human umbilical cord cells for neonatal encephalopathy in the first 14 days of life conducted at Duke University (NCT00593242).

## 2 Conclusions

Perinatal brain damage in the term infant remains an important clinical problem. Adverse outcome after severe term perinatal asphyxia is still 45%. Although many aspects of the pathophysiology of hypoxic–ischaemic brain injury have been elucidated, therapeutic hypothermia is the only neuroprotective strategy for standard care. Further studies are needed to evaluate the use of compounds that have been shown effective in animal experiments.

## Figures and Tables

**Table 1:** Examples of neuroprotective treatment in animal models of hypoxia–ischemia.

Experimental model	Treatment	Findings
Anti-apoptotic/mitoprotective agents		
Hypoxia–ischaemia in 7 day old rats (Cheng et al., 1998)	Boc-aspartyl(OMe)-fluoro-methylketone (broad spectrum caspase inhibitor) given icv (100 nmol/pup) 3 h after insult	Decrease of caspase activity and lesion volumes in cortex, hippocampus and striatum; attenuation of neuronal loss in hippocampus.
Hypoxia–ischaemia in 9 day old mice (Wang et al., 2010)	BAX-inhibiting peptide (BIP; 25 μg/pup) vs*.* a BIP negative control was given icv prior to insult	BIP reduced BAX translocation to mitochondria, cytochrome C release and caspase-3 activation; BIP attenuated infarction and white matter injury assessed at 5 days and improved memory and sensorimotor functions 7 weeks following insult.
Hypoxia–ischaemia in 7 day old rats (Nijboer et al., 2011)	Pifithrin-μ (PFT-μ) (inhibitor of p53 association with mitochondria) (2–8 mg/kg ip) given 0-21 h after HI.	PFT-μ(8 mg/kg) reduced mitochondrial permeabilisation, caspase activation and tissue loss evaluated both at 2 days and 10 weeks after HI. Improvement of cognitive and sensorimotor functions at 6–10 weeks. PFT-μ was effective if given ≤ 6 h after insult.
Ibotenate in 5 day old mice; hypoxia–ischaemia in 9 day old mice, focal ischaemia in 7 day old rats (Chauvier et al., 2011)	TRP601 (caspase-2 inhibitor) given 0.1–1 mg/kg ip at different time points after the insult.	TRP601 reaches the CNS, inhibits caspase-2/− 3 and mitochondrial cytochrome C release. Reduction of brain injury in all three models with a therapeutic window of 6 h after HI; TRP601 does not affect physiological apoptosis or CNS development and has a favourable safety profile in rodents and canine neonates.

Immuno-modulatory and anti-oxidative agents		
Hypoxia–ischaemia in 7 day old rats (Arvin et al., 2002)	Minocycline (tetracycline derivative having anti-inflammatory action) 22.5 or 45 mg/kg given ip before or after HI.	Tissue area loss was markedly reduced in striatum, cortex and hippocampus at 1 week after HI if drug was given before or directly after HI; minocycline attenuated apoptotic and necrotic cell death.
Umbilical cord occlusion, foetal sheep at 91–93 days of gestation (Welin et al., 2007)	Melatonin (20 mg/kg/h) given to the foetus iv starting immediately after cord occlusion and for 6 h vs*.* vehicle.	The production of 8-isoprostanes, activated microglia cells and TUNEL-positive cells following insult were attenuated in melatonin treated foetuses. There was no difference in the overall neuropathology score between groups.
Hypoxia–ischaemia in newborn piglets (Robertson et al., 2013)	Hypothermia (33.5 °C 2–26 h after HI) vs*.* hypothermia + melatonin (5 mg/kg/h) iv over 6 h	Melatonin add-on therapy increased the levels of whole brain (31)P magnetic resonance spectroscopy nucleotide triphosphate/phosphate pool; decreased TUNEL in thalamus, internal capsule, putamen and caudate, caspase-3 in thalamus and activated (CD86+) microglia.
Hypoxia–ischaemia in 7 day old rats (Jin et al., 2007, Jin et al., 2009)	Cromolyn (inhibits mast cell degranulation)(50 mg/kg) was given sc 30 min before, immediately, 1 h and 24 h after HI or only immediately, 1 h and 24 h after HI.	Cromolyn reduced the number of mast cells, degranulated mast cells and brain injury (assessed with fluoro-jade B at 48 h post-HI and neuronal loss and brain atrophy at 4 weeks) in all brain regions. The number of astroglia and microglial/macrophage CD45+ cells was attenuated.
Combined LPS + hypoxia–ischaemia in mice (Bolouri et al., 2014)	1018 (innate defence regulatory peptide) was given 8 mg/kg, ip 3 h following LPS + HI.	The 1018 peptide modulated the LPS-evoked cytokine/chemokine response to LPS in mouse microglial cultures; 1018 reduced white and grey matter injury *in vivo* and gene expression analysis showed reduction of pro-inflammatory and cell death related genes.
Hypoxia–reperfusion in newborn piglets (Liu et al., 2010)	N-acetylcysteine (150 mg/kg bolus and 20 mg/kg/h iv) for 24 h	N-acetylcysteine improved oxygen delivery and attenuated the increase in cortical caspase-3 activity and lipid hydroperoxide concentrations up to 48 h post-hypoxia. Reduced and oxidised glutathione was not affected by drug treatment.
LPS + hypoxia–ischaemia in 8-day old rats (Wang et al., 2007)	N-acetylcysteine (200 mg/kg ip) prior + directly after HI or 0 h and 24 h after HI.	N-acetylcysteine (pre + post or only post HI) reduced tissue volume loss and neuropathology score in cerebral cortex, hippocampus and thalamus. Isoprostane activation, nitrotyrosine formation, caspase-1 and -3, calpain activation were attenuated and the drug increased levels of the anti-oxidants glutathione and thioredoxin.
Hypoxia–ischaemia in 7 day old rats (Hagberg et al., 1996, Hu et al., 2005)	IL-1ra (IL-1 receptor antagonist) was given either 3.3 μg/rat icv prior to HI or 2 μg/rat icv 2 h after HI.	IL-1ra reduced brain injury (assessed as hemispheric weight deficit 2 weeks after HI), DNA fragmentation and activation of caspase-3 (in cerebral cortex and hippocampus).
Hypoxia–ischaemia in 7 day old rats (Balduini et al., 2001, Li et al., 2010)	Simvastatin (cholesterol lowering drug) was given prophylactically (20 mg/kg sc daily post-natal days 1–7) prior to HI	Attenuation of volume loss in cerebral cortex, hippocampus and whole hemisphere 10 weeks after HI. Simvastatin attenuated behavioural deficits, improved myelination (myelin-basic protein staining) inhibited microglial/macrophage activation (OX-42 positive cells) and reduced the numbers of pyknotic cells.

Trophic action, enhancement of neurogenesis/myelogenesis		
Hypoxia–ischaemia in 7 day old rats (Almli et al., 2000, Han et al., 2000)	Brain-derived neurotrophic factor (BDNF) (10 μg/pup) was administered icv prior to HI	BDNF attenuated memory and spatial memory impairments assessed 2–3 weeks after the insult and reduced injury in cortex, hippocampus, and striatum at 4 weeks; BDNF attenuated markedly caspase-3 activation after the insult.
Bilateral carotid artery occlusion in near term (121–128 days of gestation) foetal sheep (Cao et al., 2003)	Insulin-like growth factor-1 (IGF-1) was given icv 90 min either a single dose (3 or 30 μg), or 3 μg followed by 3 μg over 24 h	3 μg (but not 30 μg) of IGF-1 increased myelination (myelin basic protein + proteolipid protein mRNA) and number of oligodendroglial precursors at 4 days after ischemia. IGF-1 attenuated caspase-3 activation and increased proliferation. IGF-1 over 24 h did not provide additional benefit.
Focal ischaemia (transient middle cerebral artery occlusion) in 7 day old rats (Gonzalez et al., 2013)	Erythropoietin (EPO) (1000 U/kg) was given icv at reperfusion, 24 h, and 7 days later	Multiple doses of EPO shifted cell fate from astroglia proliferation towards neurogenesis and oligodendrogliosis at 3 and 14 days in striatum.
Focal ischaemia (transient middle cerebral artery occlusion) in 10 day old rats (Gonzalez et al., 2007)	EPO(5 units/g) was injected ip directly upon reperfusion	EPO moderately prevented hemispheric volume loss at 6 weeks after ischaemia. EPO increased the percentage of newly generated neurons while decreasing newly generated astrocytes following brain injury.
Umbilical cord occlusion (15–18 min) in term (173 days of gestation) non-human primates (*Macaca nemestrina* (pigtailed macaques) (Traudt et al., 2013)	EPO(1000 U/kg/day i.v. 30 min, 24 h, 48 h, and 7 days after asphyxia) + hypothermia (HT) (33.5 °C for 72 h after resuscitation) vs*.* only hypothermia or saline treatment	The animals were followed up for 9 months. Death or moderate-severe cerebral palsy occurred in 44% of HT and in 0% of HT + EPO treated animals. EPO improvement of motor and cognitive responses, cerebellar growth, and MRI measures. (Diffusion tensor imaging showed improved mode of anisotropy, fractional anisotropy, relative anisotropy, and volume ratio) as compared to saline-treated infants.

Anti-excitotoxic compounds		
Hypoxia–ischaemia in 7 day old rats (Hagberg et al., 1994, McDonald et al., 1987)	MK-801 (Dizocilpine) (NMDA-receptor antagonist) was given ip (0.3, 0.5, 0.75 mg and 1 mg/kg) vs. vehicle after HI	MK-801 (0.3 and 0.5 mg/kg) given after HI reduced brain injury (assessed as hemispheric weight deficit vs. contralateral); MK-801 (0.5 mg/g) reduced cortical infarct volume. A dose of 1 mg/kg given before or during HI (but not after HI) reduced hemispheric injury assessed 5 days after HI.
Bilateral carotid artery occlusion in near term (123–137 days of gestation) foetal sheep (Tan et al., 1992)	MK-801 (Dizocilpine)(0.3 mg/kg ip bolus) was given at 6 h after the insult followed by continuous infusion of 1 mg/kg over the next 36 h	The intense epileptiform activity seen in the control group during recovery was completely suppressed in the MK-801-treated group. The onset of secondary cortical edema was delayed and neuronal damage was reduced, particularly in the lateral cortex and hippocampus 3 days after insult.
Hypoxia–ischaemia in 7 day old rats (Andine et al., 1988)	Kynurenic acid (non-specific antagonist of excitatory amino acid receptors) (300 mg/kg) immediately after HI	Kynurenic acid reduced brain injury (assessed as hemispheric weight deficit vs. contralateral) 2 weeks after HI.
Hypoxiaischaemia in 7 day old rats (Hagberg et al., 1994)	NBQX (AMPA-receptor antagonist) (15 + 15 or 20 + 20 mg/kg) ip directly and 1 h after HI	NBQX (20 + 20 mg/kg) attenuated hemispheric volume loss after HI, improved morphology score and reduced the infarct volume. The lower dose did not affect outcome.
Hypoxia–ischaemia in 7 day old rats (Follett et al., 2000)	NBQX (AMPA-receptor antagonist) (20 mg/kg) ip starting directly after HI and thereafter repeated doses every 12 h for 48 h	NBQX improved myelination 4 days after HI (assessed with myelin basic protein immunoreactivity) and provided a significant preservation of O1 positive oligodendroglial precursors.
Hypoxia–ischaemia (transient occlusion of carotid arteries and hypotension) in newborn piglets (Schubert et al., 2005)	Topiramate was given as a loading dose 50 mg/kg 1 h after insult and maintenance dose 20 mg/kg/day until termination of the experiment after 72 h	Topiramate treated animals exhibited a markedly reduced amount of neuronal damage in frontal, temporoparietal and occipital cortex, striatum and hippocampus compared with vehicle-treated 3 days after HI. There was increased numbers of TUNEL positive cells in subventricular zone and frontal white matter in topiramate treated piglets.
Hypoxia–ischaemia in 7 day old rats (Noh et al., 2006)	Topiramate (20, 50, 100 mg/kg/dose ip) before and after HI or only after (directly + 2 h post HI); also peroral treatment (50 mg/kg/dose) pre + post HI was given	Topiramate given iv or perorally reduced brain damage in cortex, striatum and hippocampus 5 days after HI and attenuated cognitive impairments. Treatment was also effective if given 2 h after HI.
Hypoxia–ischaemia in 7 day old rats (Ma et al., 2005)	Xenon (20–70%) was either added during HI or 2 to 24 h after the insult for 90 min alone or in combination with HT (30–35 °C) for different durations	A combination of xenon and HT administered 4 h after HI injury in neonatal rats provided synergistic neuroprotection assessed by morphology, hemispheric weight, and by functional neurological (motor and balance) assessments up to 30 days after the injury.
Global hypoxia–ischaemia in newborn pigs (Chakkarapani et al., 2010)	Xenon (50%) for 18 h after HI in combination with normothermia or HT (33.5 °C) for 12 h or 18 h compared with no treatment or HT for 12 h or 18 h without Xenon	Combining xenon with HT provided added neuroprotection, e.g. xenon and 24 h HT offered marked histological neuroprotection in thalamus, brainstem, white matter, basal ganglia, cortical grey matter, cerebellum and hippocampus assessed 3 days after HI. Neurologic functional scores improved in the xenon + HT group.

Abbreviations: EPO – erythropoietin; HI — hypoxia–ischaemia; HT — hypothermia; icv — intracerbroventricularly; ip — intraperitoneally; iv — intravenously; LPS — lipopolysaccharide; NBQX — 6-nitro-7-sulfamoylbenzo(f)quinoxaline-2,3-dione; sc — subcutaneously.

**Table 2:** Techniques used for assessment of brain injury after perinatal hypoxia–ischaemia^a^.

	Advantages	Disadvantages
Clinical methods		
Apgar score	Used worldwide, cheap, easily applicable, high prediction of mortality in case of low score at 5 min (Bonifacio et al., 2015, Casey et al., 2001, Laptook et al., 2009, Sarkar et al., 2010)	Not specific for hypoxia–ischaemia; infection, sedative drugs, congenital abnormalities may lower Apgar scores

Clinical examination		
Sarnat score (Sarnat and Sarnat, 1976)	Widely accepted, used as entry criteria in clinical trials (Shankaran et al., 2005)	Based on a limited number of patients; consists of a combination of clinical assessment and EEG, not specific for hypoxia–ischaemia
Thompson score (Thompson et al., 1997)	Does not require expensive technology, easily applicable	Not specific for hypoxia–ischaemia, not widely accepted

Biochemical methods		
(Umbilical cord) blood gas values (Bonifacio et al., 2015, Wayock et al., 2014)	Excellent information on hypoxia-induced metabolic acidosis, widely accepted	Need to be performed rapidly after birth No direct relation with severity or extent of brain injury
Biomarkers(S100b, NSE) (Massaro et al., 2012, Serpero et al., 2013, Tonni et al., 2014)	Easily applicable	No direct relation with localization of brain injury

Electrophysiological methods		
Evoked potentials (Eken et al., 1995, van Laerhoven et al., 2013)	Good predictive value after asphyxia	Not easily applicable, technically difficult, not specific for hypoxia–ischaemia
Amplitude-integrated EEG (Thoresen et al., 2010, Toet et al., 1999, van Laerhoven et al., 2013)	Continuous information of presence of seizures and background activity; excellent predictive value after perinatal asphyxia	Requires training of neonatal staff Not specific for hypoxia–ischaemia

Near-infrared spectroscopy		
NIRS (Lemmers et al., 2013)	Good predictive value after asphyxia	Not easily applicable; only trend monitor; limited experience

Neuroimaging		
Cranial ultrasound (Eken et al., 1994)	Bedside technique, excellent tool for diagnosis of major haemorrhage or changes in basal ganglia/thalamus after asphyxia; can be performed repeatedly	Ischaemic changes can be seen after 2 days; requires training of neonatal/radiology staff
MRI (Alderliesten et al., 2011, Rutherford et al., 2010)	Excellent predictive value after asphyxia; essential to detect other causes of neonatal encephalopathy (ischaemic stroke, sinovenous thrombosis, infections, metabolic causes, congenital malformations)	Not easily applicable; requires extensive training of neonatal/radiology staff; optimal timing not yet established (in particular after therapeutic hypothermia); impossible in most severe cases due to clinical limitations
Proton MR spectroscopy^b^ (Alderliesten et al., 2011, Thayyil et al., 2010)	Excellent predictive value after asphyxia	Not easily applicable; requires extensive training of neonatal/radiology staff; impossible in most severe cases due to clinical limitations

a Most techniques were studied prior to the era of therapeutic hypothermia; hypothermia may change the predictive value of these techniques (Sabir and Cowan, 2015).

b Not an imaging technique, but data are obtained during the same session as MRI.
